# Mannosylated Lipoarabinomannans from *Mycobacterium Avium* Subsp. Paratuberculosis Alters the Inflammatory Response by Bovine Macrophages and Suppresses Killing of *Mycobacterium* Avium Subsp. *Avium* Organisms

**DOI:** 10.1371/journal.pone.0075924

**Published:** 2013-09-30

**Authors:** Cleverson Souza, William C. Davis, Torsten M. Eckstein, Srinand Sreevatsan, Douglas J. Weiss

**Affiliations:** 1 Department of Veterinary Clinical Sciences, Washington State University, Pullman, Washington, United States of America; 2 Department of Veterinary Microbiology and Pathology, Washington State University, Pullman, Washington, United States of America; 3 Department of Microbiology, Immunology, and Pathology, Colorado State University, Fort Collins, Colorado, United States of America; 4 Department of Veterinary Population Medicine, University of Minnesota, St. Paul, Minnesota, United States of America; 5 Department of Veterinary and Biomedical Sciences, University of Minnesota, St. Paul, Minnesota, United States of America; Centre National de la Recherche Scientifique - Université de Toulouse, France

## Abstract

Analysis of the mechanisms through which pathogenic mycobacteria interfere with macrophage activation and phagosome maturation have shown that engagement of specific membrane receptors with bacterial ligands is the initiating event. Mannosylated lipoarabinomannan (Man-LAM) has been identified as one of the ligands that modulates macrophage function. We evaluated the effects of Man-LAM derived from *Mycobacterium avium* subsp. *paratuberculosis* (*MAP*) on bovine macrophages. Man-LAM induced a rapid and prolonged expression of IL-10 message as well as transient expression of TNF-α. Preincubation with Man-LAM for up to 16 h did not suppress expression of IL-12 in response to interferon-γ. Evaluation of the effect of Man-LAM on phagosome acidification, phagosome maturation, and killing of *Mycobacterium avium* subsp. *avium (MAA)* showed that preincubation of macrophages with Man-LAM before addition of *MAA* inhibited phagosome acidification, phagolysosome fusion, and reduced killing. Analysis of signaling pathways provided indirect evidence that inhibition of killing was associated with activation of the MAPK-p38 signaling pathway but not the pathway involved in regulation of expression of IL-10. These results support the hypothesis that *MAP* Man-LAM is one of the virulence factors facilitating survival of *MAP* in macrophages.

## Introduction

The immune response to mycobacterial infection involves phagocytosis of bacteria by mononuclear phagocytes and sequestration within phagosomes [1]–[3]. Pathogenicity of mycobacteria appears to depend on the capacity of the bacterium to prevent macrophage activation and phagosome maturation and to attenuate induction of a Th1 immune response [3], [4]. The mechanisms by which mycobacteria interfere with macrophage antimicrobial mechanisms are complex but primarily involve initiation of cell signaling pathways through interaction with cell membrane receptors, blocking phagosome acidification and phagolysosome fusion, and attenuating presentation of bacterial antigens to the immune system [2], [5]. We have studied the interaction of *Mycobacterium avium* subsp. *paratuberculosis* (*MAP*), with bovine macrophages [6]–[10]. *Mycobacterium avium* subsp. *paratuberculosis* is the causative agent of paratuberculosis, a chronic progressive enteritis in ruminants [6]. In vitro, *MAP* -infected macrophages rapidly phosphorylate Mitogen Activated Protein Kinase-p38 (MAPK-p38), stimulate expression of IL-10, and fail to acidify phagosomes and interfere with killing of *MAP* [6]–[10]. Results of a variety of blocking studies indicate that *MAP*-induced activation of MAPK-p38 is a major mechanism involved in suppression of antimicrobial responses within phagocytes. MAPK-p38 activation initiates production of the anti-inflammatory cytokine IL-10 and may be involved in inhibiting phagosome acidification by interfering with important phagosome effector molecules [9], [10].

Mannosylated lipoarabinomannan (Man-LAM) is a major mannose-capped lipoglycan cell wall component of pathogenic mycobacteria [11]. It is a virulence factor in several virulent mycobacteria that affects dendritic cell and macrophage function [11]. In contrast, nonpathogenic mycobacteria contain phosphoinositol capped LAMs (PILAMs) that do not have the same effect on DC and macrophage function. Man-LAM, derived from *Mycobacterium tuberculosis*, has been shown to interact with mannose receptors on human macrophages, whereas PILAM preferentially binds to CD14 [12], [13]. PILAM was shown to induce higher levels of pro-inflammatory mediators compared to Man-LAM [14]. These findings suggest that Man-LAM from *MAP* uses the same receptor and may be involved in activation of the MAPKp38 pathway, modulation of IL-10 secretion, phagosome acidification, and bacterial killing. The present study was conducted to investigate this possibility.

## Materials and Methods

### Bacterial strain and culture conditions

*Mycobacterium avium* subsp. *avium* (*MAA*) strain 35716 was obtained from the American Type Culture Collection. This strain was isolated from a naturally-infected cow. Bacteria were grown to a concentration of approximately 10^8^/ml, washed, and re-suspended in broth medium (OADC, Difco Labs, Detroit, MI), Tween 80 (Sigma Chemical Co, St. Louis, MO), and 5% fetal bovine serum (Allied Monitor Inc., Fayette, MO) as previously described [9]. Viability of bacteria was determined by propidium iodide (Calbiochem, La Jolla, CA) exclusion.

### Chemical inhibitors and antibodies

SB203580 (10 µM, Calbiochem, La Jolla, CA) was used as a specific inhibitor of MAPK-p38. A polyclonal goat anti-human IL-10 antibody (clone AF-177-NA, R&D Systems, Minneapolis, MN) was used to neutralize IL-10 activity. This antibody has been previously reported to neutralize bovine IL-10 [6]. Lysosomes were labeled with mouse anti-bovine CD63 and associated conjugated antibody (clone CC25, Serotec USA, Washington, DC).

### Preparation of Man-LAM from MAP K-10 strain

*Mycobacterium avium* subsp. *paratuberculosis* K-10 cells were re-suspended in breaking buffer consisting of 4% triton ×114 in PBS supplemented with pepstatin, leuptin, PMSF, DNAse I, and RNAse I and subjected to bead beating to destroy clumps. The suspended cells were then sonicated followed by centrifugation at 27,000× g at 4°C for 1 hr. Supernatant was saved for further extraction. The pellet was re-suspended in breaking buffer and was centrifuged again. The supernatant was removed and combined with the first supernatant. Man-LAM was extracted from supernatant by the Triton ×114 method. Equal volumes of PBS and Triton ×114 were used for bi-phasic separation. Each phase was then transferred to new tubes. Man-LAM was precipitated from the detergent layer with 10 volumes of ice-cold acetone for 48 hours. Precipitate was collected by centrifugation and the resulting pellet was air-dried. The dry pellet was suspended in PBS and an equal volume of buffered phenol was added. The solution was mixed for 30 minutes. Bi-phasic separation was achieved by incubation on ice for 30 minutes followed by centrifugation. To each phase, equal volumes of the opposite solution were added and extraction was performed twice. All aqueous layers were combined and dialyzed against water to remove remaining phenol and salt. Purified Man-LAM was then lyophilized for storage.

### Macrophages Isolation and Culture

This study was carried out in strict accordance with the recommendations in the Guide for the Care and Use of Laboratory Animals of the National Institutes of Health. The protocol was approved by the Committee on the Ethics of Animal Experiments of the University of Minnesota. Blood was collected from 3 healthy adult Holstein cows that tested negative for paratuberculosis as determined by culture of fecal samples and serum ELISA tests [14]. Mononuclear cells were isolated by use of Percoll (Sigma Chemical Co, St. Louis, MO) density gradient centrifugation and cultured, as described [9], [10]. Isolated cells were washed in Dulbecco's PBS solution and re-suspended at 1×10^7^ mononuclear cells/ml in RPMI 1640 medium containing 10% fetal bovine serum. *MAA* were added to cultures of macrophages (MOI: 10 bacilli/macrophage) and incubation was continued at 37°C and 5% CO_2_.

### Determination of IL-10, IL-12, and TNF-α gene expression by RT-PCR

The mRNA was harvested from macrophages using a commercial kit (RNeasy Kit, Qiagen, Valencia, CA). Integrity of RNA preparations was assessed by use of RNA agarose gel electrophoresis. Genomic DNA was removed from mRNA samples by use of a commercial kit (DNA-Free™, Calbiochem Inc., La Jolla, CA), following the manufacturer instructions. IL-10, IL-12p40, and TNF-α gene expression was determined by RT-PCR as described [8]. Results were expressed as relative fold expression using the Delta Ct method [15]. GAPDH expression was used to normalize the results. Results showed no variation in the expression of GAPDH in *MAA*-infected macrophages with or without Man-LAM treatment when compared to untreated macrophages (data not shown) Incubation of monocytes with Man-LAM with or without polymyxin B or DMSO did not alter *IL-10, IL-12p40, and TNF-α gene expression by RT-PCR* (data not shown).

### IL-10 ELISA

Culture supernatants were harvested at 6 or 24 h after addition of bacteria. Cytokine sandwich ELISA was used to detect IL-10 as previously described [9], [16]. A bovine IL-10 protein standard (gift from Dr. Chris Howard, Institute of Animal Health, Berkshire, UK) was serially diluted and added to the corner wells.

### Phagosome acidification

Acidification of *MAA*-containing phagosomes was determined by use of a fluorescence technique as previously described [6]–[10]. In brief, bacteria were labeled with fluorescein isothiocyanate and then incubated with monocyte derived macrophages grown on 22×22-mm coverslips (MOI 10∶1) for 18 h. Biotracker Red (a fluorescent stain which is taken up by acidified phagosomes) was added (50 nM) during the last 30 minutes of incubation. Coverslips were inverted onto a glass slide and then examined with a confocal microscope. Intensity of green and red fluorescence was sequentially recorded at 0.5 µM increments through the depth of the cells. Data were analyzed with an image-analysis program. Sequential images were merged. The intensity of green and red fluorescence of at least 100 phagosomes containing mycobacteria was quantified in 3 separate experiments. The results were reported as a co-localization coefficient. The co-localization coefficient was defined as the density of red fluorescence divided by the density of green fluorescence to obtain a numerical value. Controls consisted of macrophages incubated with unlabeled bacteria with or without Lysotracker Red, and macrophages incubated with labeled organisms without addition of Lysotracker Red.

### Phagosome-lysosome fusion

LAMP 3 (lysosome-associated membrane protein 3, CD63) co-localization on bacteria-containing phagosomes was used as a marker (conjugated with phycoerythrin B) to establish phagosome-lysosome fusion as previously described [9], [10]. The intensity of green and red fluorescence of at least 100 phagosomes containing mycobacteria were quantified. The co-localization coefficient was used as described above to obtain a numerical value. Controls included macrophages incubated with bacteria and isotype-matched control antibody.

### Phagocytosis and intracellular survival of mycobacteris

Macrophages were incubated with *MAA* bacteria (MOI 10∶1) for 30 or 60 min before staining with Ziehl-Neelsen carbolfuchsin stain, (Sigma Chemical CO, St. Louis, MO). The percentages of macrophages containing bacteria were determined by counting a minimum of 200 cells by use of light microscopy. Killing of the bacteria was assessed by use of a live-dead stain, (BackLight kit, Invitrogen Inc., Carlsbad, CA) as described for mycobacteria [9], [10]

### Apoptosis

Bovine macrophages were washed and Hoechst 33342 stain (Invitrogen Inc., Carlsbad, CA) was added for 10 min and macrophages were examined by use of a fluorescent microscope (Nikon E800, Melville, NY) with excitation at 350 nm and emission at 461 nm [17]. At least 200 cells were counted and the percentage of fluorescent cells was determined.

### Statistical analysis

All tests were done in duplicate or triplicate and results of at least three separate experiments were evaluated. Results were expressed as mean ± SD. Differences between cell cultures incubated with and without addition of inhibitors were analyzed by use of the paired student *t*-test. P<0.05 was considered statistically significant.

## Results

### Effects of Man-LAM on IL-10, IL-12p40, and TNF-α expression by bovine macrophages

**Figure 1:**
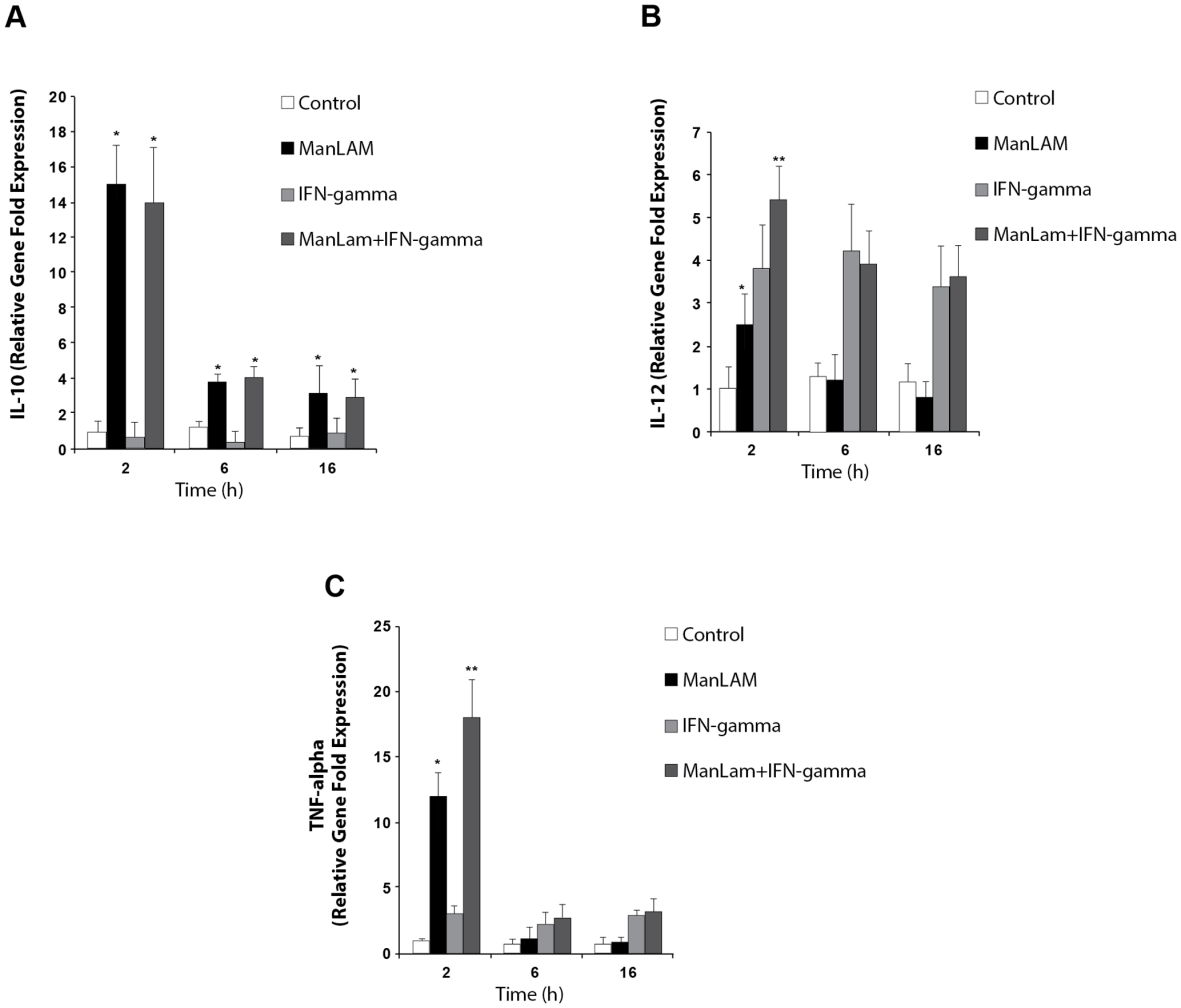
Interleukin-10 (IL-10, A) IL-12p40 (B), and tumor necrosis factor-alpha (TNF-alpha, C) mRNA expression by bovine macrophages in response to mannosylated lipoarabinomannans (Man-LAM, 5 µg/ml) derived from *Mycobacterium avium* subsp. *paratuberculosis* and or interferon-gamma (IFN-gamma). Macrophages were incubated with or without Man-LAM for 2, 6, or 16 h and then incubated with or without IFN-gamma for 2 h. Similar results were achieved with doses of 1 and 10 µg/ml. Asterisks indicate statistically significant differences between treated and untreated groups using the Student's t-test. Double asterisk indicates statistically significant differences between macrophages treated with Man-LAM + IFN-gamma and those treated with either Man-LAM or IFN-gamma alone using the Student's t-test. *P<0.05* was considered statistically significant.

Macrophages were incubated with or without Man-LAM for 2, 6, and 16 h at concentrations of 1, 5, or 10 µg/ml in the presence of polymyxin B to neutralize any lipopolysaccharide contamination. Cytokine expression was determined. Incubation of macrophages at all 3 concentrations of Man-LAM for 2 h resulted in a 15-fold increase in IL-10 mRNA expression, a 12-fold increase in TNF-*α* expression, but only a slight increase in IL-12p40 expression (Fig. 1). Expression of IL-10 at 6 and 16 h was greater than control but expression of IL-12p40 and of TNF-*α* were similar to the control for all concentrations of Man-LAM. Expression of IL-10 was confirmed by determination of IL-10 protein concentration in culture supernatants from macrophages treated with 5 µg/ml of Man-LAM for 6 h (74±0.04 pg/ml) compared to control macrophages (14±0.02 pg/ml).

The capacity of Man-LAM to suppress cytokine expression by macrophages in response to recombinant bovine interferon-γ (IFN-γ) was evaluated. Macrophages were incubated with or without Man-LAM at a concentration of 5 or 10 µg/ml for 2, 6, or 16 h. Thereafter, IFN-γ (10 U/ml) was added, incubation was continued for 2 h. IL-10, IL-12p40, and TNF-*α* expression was determined. IFN-γ treatment alone failed to stimulate IL-10 expression. It did, however, induce a 4-fold increase in IL-12p40 expression at 2, 6, and 16 h and a modest increase in TNF-α expression (Fig. 1). Preincubation with Man-LAM for up to 16 h did not suppress IL-12p40 expression in response to IFN-γ (Fig. 1B).

### Effect of Man-LAM on survival of bacteria

**Figure 2:**
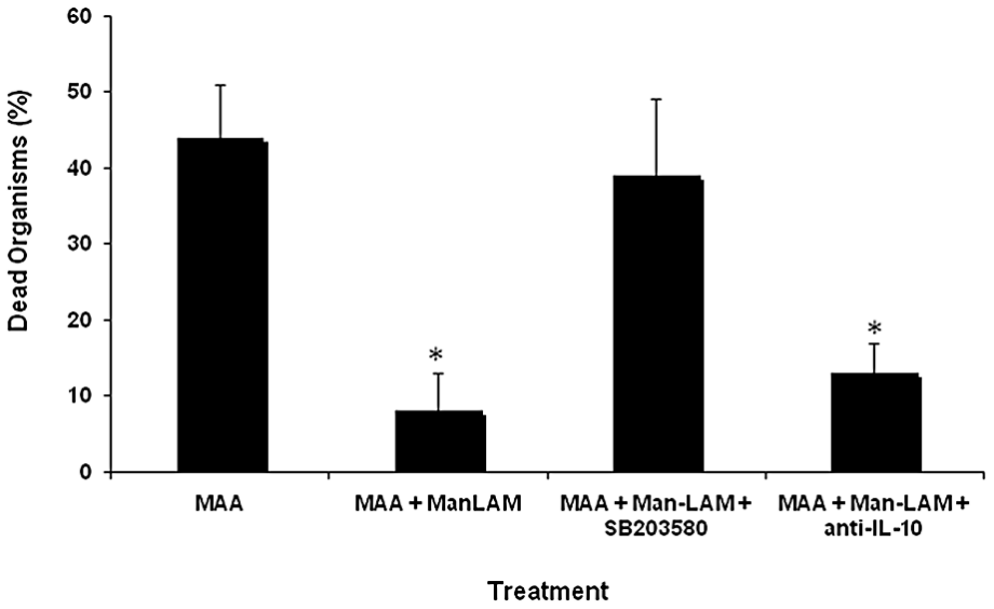
Effects of mannosylated lipoarabinomannan (Man-LAM) on killing of *Mycobacterium avium* subsp. *avium* by bovine macrophages. Bovine macrophages (1×10^6^/ml) were incubated with or without Man-LAM (1 µg/ml) for 2 h and then were incubated with or without *Mycobacterium avium* subsp. *avium* for 72 h. The percentage of dead bacteria was determined by use of a fluorescent live-dead stain. The effects of IL-10 and mitogen-activated protein kinase-p38 (MAPK-p38) on Man-LAM-mediated inhibition of killing were determined by pre-incubating macrophages with a neutralizing anti-IL-10 antibody and SB203580 (i.e., specific MAPK-p38 inhibitor) respectively. Asterisks indicate statistically significant differences between macrophages treated with Man-LAN compared to those not treated with Man-LAM and between macrophages treated with inhibitor + Man-LAM and macrophages treated with Man-LAM alone using the Student's t-test. *P<0.05* was considered statistically significant.

Previous studies have shown that macrophages have the capacity to kill *MAA* but showed a limited capacity to kill *MAP* in vitro. To evaluate the effect of Man-LAM on mycobacterial survival within bovine macrophages, macrophages were incubated with *MAA* with or without preincubation with Man-LAM for 2 h. As in previous studies, incubation of macrophages with *MAA* resulted in killing of 40% to 50% of the bacteria after 72 h of incubation (Fig. 2). Alternatively, when macrophages were preincubated with 5 µg/ml of Man-LAM for 2 h, killing was reduced to 8±5%. The Man-LAM-induced reduction in killing was enhanced by addition of the MAPK-p38 inhibitor, SB203580, but not by addition of a saturating concentration of anti-bovine IL-10 antibody.

To determine if the reduced killing of *MAA* in cultures incubated with Man-LAM was the result of reduced phagocytosis, we evaluated the percentage of cells phagocytizing bacteria after staining with Ziehl-Neelsen carbolfuchsin stain. The percentage of macrophages containing bacteria at 30 min with and without addition of Man-LAM (5 µg/ml) was 74±7% and 72±5% respectively and, at 60 min, was 85±6% and 88±7% respectively. Very few free bacteria were observed at 60 min under either culture condition. To evaluate macrophage viability in cultures incubated with and without Man-LAM, we measured apoptosis at 24 and 48 h of incubation. The percentage of apoptotic cells in macrophages incubated with *MAA* alone for 24 h or 48 h was 18±4% and 27±11% respectively. Macrophages incubated with *MAA* and Man-LAM for 24 h or 48 h had 15±3% and 19±5% apoptotic cells respectively.

### Effects of Man-LAM on phagosome acidification

**Figure 3:**
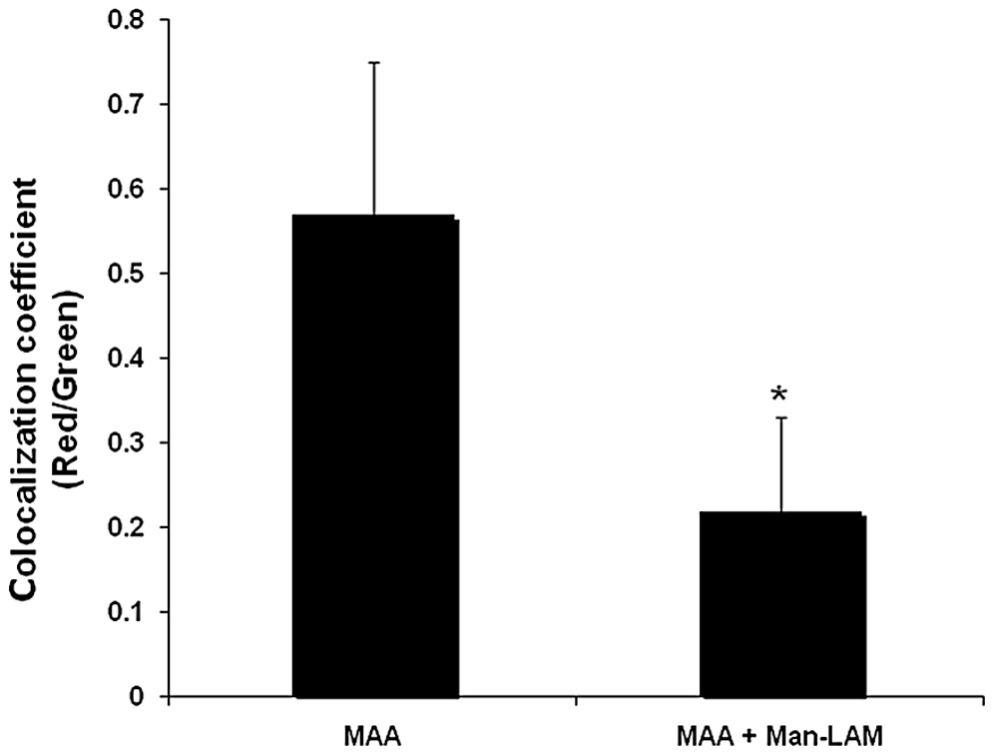
Effects of mannosylated lipoarabinomannan (Man-LAM) on acidification of phagosomes containing *Mycobacterium avium* subsp. *avium* . Bovine macrophages (1×10^6^/ml) were incubated with or without Man-LAM (5 µg/ml) for 2 h and then were incubated with *Mycobacterium avium* subsp. *avium* for 18 h. The ratio of red (i.e., acidified phagosome) to green fluorescence (i.e., non-acidified phagosome) of at least 100 phagosomes was determined by use of confocal microscopy. Asterisk indicates statistically significant difference between the treated and untreated group using the Student's t-test. *P<0.05* was considered statistically significant.

Confocal microscopy was used to quantify phagosome acidification. Highly acidified phagosomes appeared yellow and nonacidified phagosomes appeared dark green. Acidification was quantified by determining the percentage of acidified (i.e. yellow) phagosomes and the colocalization of the red and green signal within the phagosome as described in methods to obtain a numerical co-localization coefficient fluorescence score. Macrophages incubated with *MAA* alone had a higher percentage of acidified phagosomes (9±6%) and a higher red to green colocalization coefficient when compared to macrophages preincubated with Man-LAM before addition of *MAA* (0.8±1.2%, p<0.01, Fig. 3).

### Effects of Man-LAM on phagolysosome fusion

**Figure 4:**
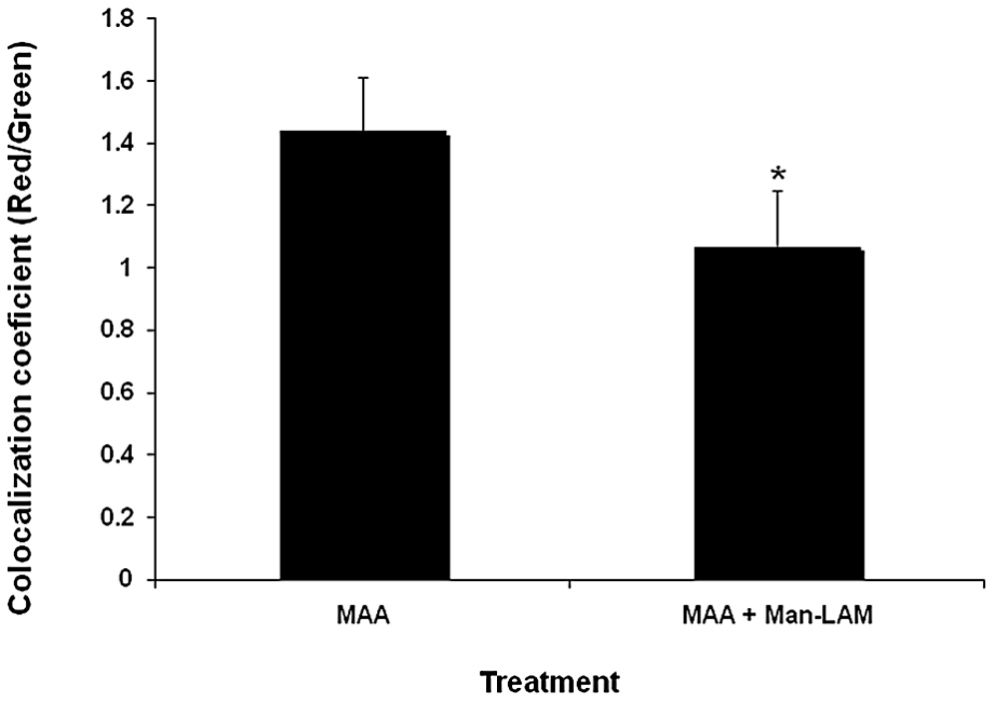
Effects of mannosylated lipoarabinomannan (Man-LAM) on phagosome-lysosome fusion of phagosomes containing *Mycobacterium avium* subsp. *avium*. Bovine macrophages (1×10^6^/ml) were incubated with or without Man-LAM (5 µg/ml) for 2 h and then were incubated with *Mycobacterium avium* subsp. *avium* for 4 h. The intensity of red (i.e., CD63; acidified phagosome) and green fluorescence (i.e., labeled) was determined by use of confocal microscopy and results were reported as a red/green colocalization coefficient. Asterisk indicates statistically significant difference between the treated and untreated group using the Student's t-test. *P<0.05* was considered statistically significant.

The effects of Man-LAM on phagosome-lysosome fusion for phagosomes containing *MAA* were evaluated by confocal microscopy. *Mycobacterium. avium* subsp. *avium* preincubated with Man-LAM had lower CD63 co-localization on the phagosome membrane when compared to *MAA* not preincubated with Man-LAM (p<0.05, Fig. 4).

## Discussion

Pathogenic mycobacteria have developed highly specialized mechanisms for survival within mononuclear phagocytes [1]–[3]. These mechanisms involve, at least in part, development of molecules that modulate the immune response [11]. Our laboratory has focused on identifying differences in how macrophages respond to *MAP* and *MAA* with the intent of gaining insight into unique host-pathogen interactions that determine pathogenicity [7], [9], [10]. We were particularly interested in obtaining information that would lead to an understanding of the mechanisms of pathogenesis at the molecular level. Previous studies showed that monocyte-derived macrophages incubated with *MAP* expressed high levels of IL-10 message), low levels of TNF-α and IL-12 message, and failed to acidify phagosomes or kill *MAP* [7], [9], [10]. In addition, we obtained indirect evidence that exposure to *MAP* activates the MAPK p38 pathway. Use of a specific chemical inhibitor blocked phosphorylation and the downstream effects of *MAP* on phagosome acidification and associated killing of bacteria. In contrast, *MAA-*infected bovine macrophages expressed less IL-10 and higher levels of TNF-α, partially acidified phagosomes, and killed approximately half the bacteria within 96 h [7], [9], [10]. Addition of a neutralizing anti-IL-10 antibody, chemically inhibiting MAPK-p38 phosphorylation, or blocking cell membrane TLR2 receptors before addition of *MAP* to macrophages, increased phagosome acidification and killing [6], [8], [10]. These data implicate IL-10 as a mediator of *MAP* survival in macrophages and provided evidence that indicate the TLR2-MAPK-p38 signaling pathways are involved in suppression of antimicrobial responses.

The exact mechanisms involved in suppression are complex and probably attributable to multiple factors. Data from studies with *Mycobacterium tuberculosis* (*Mtb*) suggest that Man-LAM is one of the factors involved in modulating the function of macrophages. Man-LAM is a major virulence factor for pathogenic mycobacteria. Man-LAMs appear to be anti-inflammatory. Studies have shown they suppress lipopolysaccharide-induced TNF-α and IL-12 production in human and mouse macrophages and dendritic cells [18]–[20]. Likewise, Man-LAM from *Mtb* has been shown to suppress oxygen radical and nitric oxide generation. [21]. Other major effects attributed to Man-LAM derived from *Mtb* include inhibition of macrophage apoptosis and inhibition of phagosome-lysosome fusion [19], [22]. Although some differences were observed in the present study, the findings provide evidence that *MAP-*derived Man-LAM is also a virulence factor with anti-inflammatory activity.

In the present study, Man-LAM induced marked increase in IL-10 message as well as secretion of IL-10. It also induced a transient increase in expression of the pro-inflammatory cytokine TNF-α at 2 h. The explanation for this difference from the effects of other Man-LAMs is not clear. However, we cannot rule out the possibility that our Man-LAM preparation had some residual lipomannan contamination. Lipomannans are the non-mannosylated biosynthetic precursor of Man-LAM and have been reported to induce TNF-α expression [24]. Man-LAM derived from *MAP* did not suppress IFN-γ-induced expression of IL-12 even at concentrations of 10 µg/ml and incubation times up to 16 h. Previous studies have documented that *Mtb* Man-LAM inhibits lipopolysaccharide-induced IL-12 expression [20], [23]. It is possible that these differences were caused by *MAP*-derived Man-LAM binding to alternative cell receptors or perhaps modulation of different cell signaling pathways. Further studies are needed to clarify the basis for these differences.

*MAP*-derived Man-LAM also had the capacity to inhibit acidification and maturation of phagosomes containing *MAA* and to attenuate bacterial killing. Preincubation of macrophages with an inhibitor of MAPK-p38 but not with anti-IL-10 attenuated the inhibitory effects of Man-LAM on bacterial killing. These data suggest that the MAPK-p38 signaling may be important in regulating phagosome maturation irrespective of its role in inducing IL-10 production. Previous studies have shown that mouse macrophage phagosomes containing *Mtb* fail to mature as indicated by reduced recruitment of the phagosomal membrane-tethering molecule early endosomal autoantigen 1 (EEA1) [25]. Addition of a chemical inhibitor of MAPK-p38 induced phagosome acidification and markers of phagosome maturation including acquisition of EEA1, LAMP 3, and lysobisphosphatidic acid [25].

The observation that Man-LAM inhibited killing of *MAA* is significant because a recent study has questioned the importance of Man-LAM as a virulence factor in mycobacteria [26]. Capless mutants of *Mycobacterium marinum* and *Mtb* reduced phagosome fusion in zebra fish and mice but bacterial survival was not affected. However, the present study supports previous findings. Man-LAM derived from *MAP* promoted survival of *MAA* and interfered with phagosome maturation. Therefore, these data provide support for Man-LAM as a major virulence factor in determining the capacity of mycobacteria to persist in mononuclear phagocytes.

In conclusion, we found that Man-LAM derived from *MAP* induces rapid expression of IL-10 by macrophages as well as transient expression of TNF-α. Man-LAM also interfered with phagosome maturation and killing of *MAA*. Inhibition of killing appeared to be independent of IL-10 but dependent on MAPK-p38 signaling. These results support the hypothesis that *MAP* Man-LAM is a major virulence factor for mycobacterial survival in macrophages and provide indirect evidence that the MAPK-p38 signaling pathway is involved. Further studies are now needed to detail how *MAP* Man-LAM modulates signaling.
